# Administration of inositol to a patient with bipolar disorder and psoriasis: a case report

**DOI:** 10.1186/1757-1626-3-69

**Published:** 2010-02-23

**Authors:** Konstantinos Kontoangelos, Nikolaos Vaidakis, Ioannis Zervas, Olga Thomadaki, Smaragda Christaki, Nikolaos G Stavrianeas, George N Papadimitriou

**Affiliations:** 1 Athens University Medical School, 1st Department of Psychiatry, Eginition Hospital, 74 Vas Sofias Avenue, 11528, Athens, Greece; 2 Athens University Medical School, 2nd Department of Dermatology, Attikon General Hospital, Rimini 1, 12462, Chaidari, Athens, Greece

## Abstract

**Background:**

This case report documents the effectiveness of inositol treatment on a chronic patient with bipolar disorder I and severe psoriasis. Her lithium treatment was discontinued due to psoriasis exacerbation and inositol was administered. The remarked positive effect of inositol was noted on her stable mood during the last 4 years, the absence of psoriatic lesions, which lead to an improved quality of life of the patient.

**Case presentation:**

A 62-year-old female Caucasian patient suffering from bipolar disorder, since the age of 32, presenting manic episodes when without lithium treatment. Lithium treatment caused severe exacerbation of psoriasis and was discontinued while anti-psoriatic treatment had no effect. The last 4 years the patient receives 3 gr per day of inositol alone and her mood has been stabilized while there is also a remarkable improvement on her psoriatic lesions.

**Conclusion:**

Taking into consideration the course of her bipolar disorder when lithium was discontinued previously we consider that the 4 years of follow up assessments of this patient as a satisfactory time period for concluding that inositol has been a very effective treatment, replacing lithium, for mood stabilization and psoriasis.

## Background

Lithium carbonate, the most common long term treatment for bipolar affective disorder, is known to precipitate psoriasis [1]. Exacerbation of psoriasis occurring during lithium treatment has been associated with decreased levels of inositol in the skin [2], and inositol has been used to treat the psoriasis, in conjunction with the lithium treatment, with beneficial results [3]. Inositol may alleviate symptoms of lithium induced-polydypsia via a central effect, but has no direct effect on lithium induced polyuria [4]. In addition to lowering skin inositol, lithium also reduces brain inositol levels by inhibition of inositol monophosphate [5]. Inositol, a naturally occurring isomer of glucose, is a key intermediate of phosphatidyl-inositol cycle, a second messenger system used by several noradrenergic, serotoninergic and cholinergic receptors [6]. In reference to bipolar depression inositol has been reported to have an antidepressant effect, when combined with a mood stabilizing regimen, such as lithium or antiepileptics. Decreased calcium levels have been documented as a property of psoriatic keratinocytes. Low calcium is believed to play a role in dysregulation of keratinocyte proliferation leading to psoriasis. The mechanism for lithium and propranolol inducing and exacerbating psoriasis has been linked to their effects of decreasing intracellular cAMP levels in the skin [7]. We present a case of a bipolar patient in whom lithium treatment was discontinued due to a severe psoriatic exacerbation. With the administration of inositol, the skin condition significantly improved, while the patient's mood remained stable, despite the absence of mood stabilizing agents.

## Case presentation

The patient is a 62-year-old female, Caucasian of Greek ethnicity, 80 kg, and 1.67 m of height. From her medical history the patient has an appendectomy at the age of 25, a miscarriage at 13 weeks of gestation when she was 26 years old, and a natural delivery at the age of 29. She is a heavy smoker the last 30 years consuming 40 cigarettes daily. There are no alcohol and substance misuse issues with the patient. There is no psychiatric history in her family of origin or current family. Her mother is treated for hypertension since the age 65, while her father died of stroke at the age of 70. At this moment the patient is under treatment with Quetiapine 100 mg (1-0-1), Mirtazapine 30 mg (0-0-1), Lorazepam 2.5 mg (1/2-0-1), Inositol 500 mg (2-2-2). Her first major depressive episode occurred in her early twenties. At the age of 32, she manifested her first manic episode and lithium treatment was initiated (lithium carbonate 300 mg t.i.d., blood level 0.85 meq/L). She remained stable on lithium for the next 8 years. At that time the patient developed psoriasis and her lithium treatment was discontinued.

**Figure 1:**
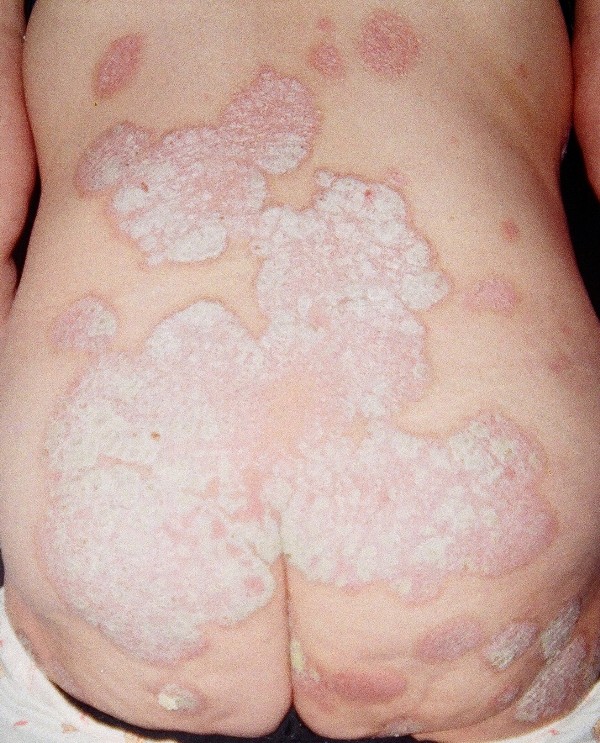
**Psoriatic lesions in dorsal areas and haunch before inositol treatment**.

**Figure 2:**
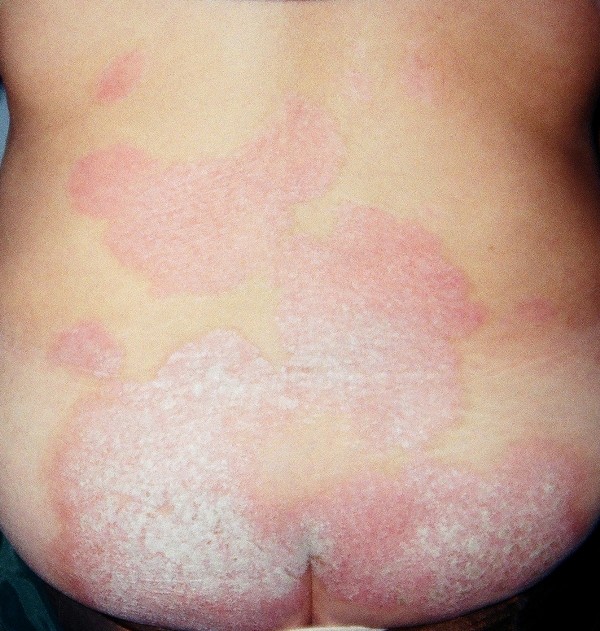
**Psoriatic lesions in dorsal areas and haunch after one month of inositol treatment**.

Lithium was replaced with carbamazepine at 400 mg b.i.d. (10 μg/ml) and psoriasis abated. However, the antiepileptic was discontinued as the patient experienced a new manic episode 18 months after the onset of carbamazepine treatment, with many residual depressive symptoms following the resolution of the manic episode. The patient requested to receive lithium again and treatment was begun with Lithium sulphate (660 mg b.i.d., blood level, 0.8), which she received for the next sixteen years until the age of 58. During this period her mood remained stable but her psoriasis progressed, covering extended areas of her skin, and specifically on the abdominal, anterior femoral, dorsal areas and haunch (Figure 1). Despite treatment with methotrexate 5 mg weekly, acitretin 50 mg q.d. and cyclosporine 100 mg q.d., at different times for 6 months each her skin condition continued to deteriorate. The patient had sharply demarcated plaques of the knees, elbows, back, scalp, and buttocks as well as few on her abdomen. Her palms, soles and nails were spared. The lesions consisted of a glossy homogeneous erythema covered with noncoherent silvery scales. Upon mechanical removal of scales by scraping, small blood droplets appeared on the erythematous surface (Auspitz sign). The exanthema was asymptomatic. Lithium was discontinued as suggested by the dermatology consultant. Two weeks after lithium discontinuation, inositol was administered to the patient, at a total daily dosage of three grams, as a sole treatment. Psoriasis improved significantly within the next month (Figure 2). She remains on inositol alone for the past four years and her mood has been satisfactorily stable, without further exacerbations of the skin condition and without any other side effects.

## Conclusion

Taking into consideration the course of her bipolar disorder when lithium was discontinued previously we consider that the 4 years of follow up assessments of this patient as a satisfactory time period for concluding that inositol has been a very effective treatment, replacing lithium, for mood stabilization and psoriasis. Given the fact that lithium is first-line agent for treatment of bipolar disorder and the seemingly benign nature of inositol supplementation, it may be useful treatment option for patients experiencing the onset of worsening of psoriasis with lithium treatment [7]. The potential of inositol as a sole treatment for psoriasis could be also investigated. Finally the mood stabilizing effects of inositol without the severe side effects of lithium should encourage further research on inositol treatment.

## Patient's perspective

The patient during our last meeting declared "I feel better, my mood is stable, I sleep well and can enjoy my daily activities, and feel happy because I don't need to take many medications for psoriasis, and have monthly blood tests for lithium levels".

## Consent

Written informed consent was obtained from the patient for publication of this case report and accompanying images. A copy of the written consent is available for review from the journal's Editor-in-Chief.

## Competing interests

The authors declare that they have no competing interests.

## Authors' contributions

KK was the doctor responsible for the patient when she was hospitalized, and had the general inspection of inositol treatment, had the idea for this case report and is the main author of this case report. NV was the head of the Department where the patient was hospitalized and supervised KK, OT and SC were the clinical/counseling psychologist responsible for the psychotherapy and support for the patient throughout her hospitalization and the last four years. IZ contributed to the write up of the present case report. NS is the dermatologist treating the patient and provided all the information on her psoriasis. GP chairman of the 1^st ^Department of Psychiatry, is the personal doctor of the patient for the last 15 years and has provided guidance to our team as a specialist on Bipolar Disorder. All authors have read and approved the final manuscript.
